# The Evolution of Ecological Diversity in *Acidobacteria*

**DOI:** 10.3389/fmicb.2022.715637

**Published:** 2022-02-02

**Authors:** Johannes Sikorski, Vanessa Baumgartner, Klaus Birkhofer, Runa S. Boeddinghaus, Boyke Bunk, Markus Fischer, Bärbel U. Fösel, Michael W. Friedrich, Markus Göker, Norbert Hölzel, Sixing Huang, Katharina J. Huber, Ellen Kandeler, Valentin H. Klaus, Till Kleinebecker, Sven Marhan, Christian von Mering, Yvonne Oelmann, Daniel Prati, Kathleen M. Regan, Tim Richter-Heitmann, João F. Matias Rodrigues, Barbara Schmitt, Ingo Schöning, Marion Schrumpf, Elisabeth Schurig, Emily F. Solly, Volkmar Wolters, Jörg Overmann

**Affiliations:** ^1^Department of Microbial Ecology and Diversity Research, Leibniz-Institute DSMZ-German Collection of Microorganisms and Cell Cultures, Braunschweig, Germany; ^2^Department of Ecology, Brandenburg University of Technology Cottbus-Senftenberg, Cottbus, Germany; ^3^Soil Biology Department, Institute of Soil Science and Land Evaluation, University of Hohenheim, Stuttgart, Germany; ^4^Bioinformatics Group, Leibniz-Institute DSMZ-German Collection of Microorganisms and Cell Cultures, Braunschweig, Germany; ^5^Institute of Plant Sciences, University of Bern, Bern, Switzerland; ^6^Microbial Ecophysiology Group, Faculty of Biology/Chemistry, University of Bremen, Bremen, Germany; ^7^Biodiversity and Ecosystem Research Group, Institute of Landscape Ecology, University Münster, Münster, Germany; ^8^Institute of Agricultural Sciences, ETH Zürich, Zurich, Switzerland; ^9^Institute of Landscape Ecology and Resources Management, University of GieBen, GieBen, Germany; ^10^Institute of Molecular Life Sciences and Swiss Institute of Bioinformatics, University of Zurich, Zurich, Switzerland; ^11^Geoecology, University of Tübingen, Tübingen, Germany; ^12^Department for Biogeochemical Processes and Biogeochemical Integration, Max-Planck Institute for Biogeochemistry, Jena, Germany; ^13^Tierökologie, Justus-Liebig-Universität, GieBen, Germany; ^14^Microbiology, Faculty of Life Sciences, Technische Universität Braunschweig, Braunschweig, Germany

**Keywords:** evolution, ecological diversity, adaptation, *Acidobacteria*, optimum niche modeling, 16S rRNA gene transcripts, physiological traits

## Abstract

Acidobacteria occur in a large variety of ecosystems worldwide and are particularly abundant and highly diverse in soils. In spite of their diversity, only few species have been characterized to date which makes *Acidobacteria* one of the most poorly understood phyla among the domain Bacteria. We used a culture-independent niche modeling approach to elucidate ecological adaptations and their evolution for 4,154 operational taxonomic units (OTUs) of *Acidobacteria* across 150 different, comprehensively characterized grassland soils in Germany. Using the relative abundances of their 16S rRNA gene transcripts, the responses of active OTUs along gradients of 41 environmental variables were modeled using hierarchical logistic regression (HOF), which allowed to determine values for optimum activity for each variable (niche optima). By linking 16S rRNA transcripts to the phylogeny of full 16S rRNA gene sequences, we could trace the evolution of the different ecological adaptations during the diversification of *Acidobacteria*. This approach revealed a pronounced ecological diversification even among acidobacterial sister clades. Although the evolution of habitat adaptation was mainly cladogenic, it was disrupted by recurrent events of convergent evolution that resulted in frequent habitat switching within individual clades. Our findings indicate that the high diversity of soil acidobacterial communities is largely sustained by differential habitat adaptation even at the level of closely related species. A comparison of niche optima of individual OTUs with the phenotypic properties of their cultivated representatives showed that our niche modeling approach (1) correctly predicts those physiological properties that have been determined for cultivated species of *Acidobacteria* but (2) also provides ample information on ecological adaptations that cannot be inferred from standard taxonomic descriptions of bacterial isolates. These novel information on specific adaptations of not-yet-cultivated *Acidobacteria* can therefore guide future cultivation trials and likely will increase their cultivation success.

## Introduction

*Acidobacteria* occur globally in a wide variety of ecosystems. The phylum encompasses a phylogenetically broad diversity of bacteria that were initially assigned to 26 subdivisions (SDs; Barns et al., 2007) and more recently into 15 classes (Dedysh and Yilmaz, 2018). Currently 12,670 different 16S rRNA sequence types are available in nucleotide databases. *Acidobacteria* are particularly abundant and diverse in soils where they can constitute up to 60% of all bacteria (Fierer, 2017; Delgado-Baquerizo et al., 2018; Eichorst et al., 2018). Of the acidobacterial 16S rRNA gene sequences that have associated meta-data, 54.9% originate from soils and 20.3% from (semi-)aquatic sediments. In contrast to their high abundance and diversity, only 61 validly named species from 27 genera (in addition to two *Candidatus* genera with one species each) could be described to date. The majority of isolates are from the single class *“Acidobacteriia”* (Parte et al., 2020). The low number of available isolates is related to considerable difficulties in cultivation (Davis et al., 2011; Eichorst et al., 2011), making *Acidobacteria* one of the poorly understood phyla among the domain Bacteria (Overmann et al., 2017) and severely limits our understanding of their ecological functions.

Based on the distribution of acidobacterial 16S rRNA genes, different *Acidobacteria* classes are known to differ in their preferences for specific values of pH, organic matter content, aboveground plant diversity, or specific habitats like the plant rhizosphere (Jones et al., 2009) and may mediate central soil functions. Culture-independent molecular analyses suggest that members of SD 1 (class *“Acidobacteriia”*) degrade plant-derived and other biogenic polymers (Ivanova et al., 2020), in particular chitin, cellulose, hemicelluloses, and xylan (Banerjee et al., 2016; Belova et al., 2018; Hausmann et al., 2018; de Chaves et al., 2019), use hydrogen at atmospheric concentrations (Giguere et al., 2021), or participate in the sulfur cycle through the dissimilatory reduction of sulfite and sulfate (Hausmann et al., 2018). Physiological analysis of the few cultivated members of the *Acidobacteriacae* within SD1 has yielded supporting evidence for the presence of the corresponding extracellular enzymes (Belova et al., 2018) and high-affinity hydrogenases (Giguere et al., 2021) and some *Acidobacteriaceae* were observed to promote plant-growth *in vitro* (Kielak et al., 2016b). However, discrepancies between genomic predictions and physiological activities have also frequently been observed (Kielak et al., 2016a; Rodrigues et al., 2020). Aside from these few known functions, the ecologically relevant traits of the majority of *Acidobacteria* species have remained mostly unknown.

We assessed the active fraction of *Acidobacteria* by high throughput sequencing of cDNA generated from the extracted 16S rRNA transcripts, since (1) the cellular ribosome content is proportional to the growth rate in most bacteria, (2) the active fraction is controlled by other environmental variables rather than total community composition as sequences of dormant cells and extracellular DNA are not included in the analysis, (3) the composition in rRNA transcripts changes in response to soil management and correlates with changes in important ecosystem functions, (4) highly active and biogeochemically relevant microbial taxa may be rare or even absent from DNA-based sequence inventories of microbial communities, and (5) rRNA transcripts allow a better differentiation of the effects of environmental drivers in at least some soil environments (Vieira et al., 2020). For consistent phylogenetic analyses, the 16S rRNA amplicon transcript sequences were linked to the phylogeny of full 16S rRNA gene sequences. In order to systematically assess potential adaptations of different species and trace the evolution of the different ecological adaptations during the diversification of *Acidobacteria*, we determined the peaks of relative 16S rRNA abundances of active acidobacterial OTUs along gradients of 41 environmental variables (optimum niche modeling). Samples were obtained from 150 comprehensively characterized German grassland soils within the framework of the German Biodiversity Exploratories (Fischer et al., 2010). The variables cover diverse physicochemical soil properties, microbial biomass, and a multitude of characteristics of the accompanying plants and soil animal communities. For a subset of these grassland soils, a previous study had provided evidence for an adaptation of individual acidobacterial species to specific environmental factors (Naether et al., 2012; Foesel et al., 2014).

## Materials and Methods

### Study Sites and Soil Sampling

A total of 150 grassland plots within the German Biodiversity Exploratories (Fischer et al., 2010)^1^ were studied. The main research objective of the Biodiversity Exploratories is to examine land use affects on biodiversity and ecosystem processes in typical central European terrestrial environments. Accordingly, the sampling sites are selected to cover a broad range of different types and intensities of land use. The German Biodiversity Exploratories cover three different study regions: the biosphere reserve Schorfheide-Chorin (Brandenburg, north-eastern Germany), the national park Hainich-Dün and its surroundings in Thuringia (central Germany), and the biosphere reserve Schwäbische Alb in Baden-Wuerttemberg (south-western Germany). In each region, 50 grassland plots with different land-use intensities, soil characteristics and vegetation composition were sampled during a joint sampling campaign over a period of 14 consecutive days in May 2011. On each plot, fourteen individual soil cores were obtained along two perpendicular transects at distances of 3 m employing a split core sampler (4.8 cm diameter) from the upper 10 cm over an area of 20 × 20 m. Parallels were mixed in the field and a 10 g aliquot was transferred immediately to liquid nitrogen and stored until extraction of RNA. For all other analyses, plant debris, coarse roots and pebbles were removed and the soil was passed through a 2 mm mesh sieve.

We compared our results to the results from a previous study termed SCALEMIC (Richter-Heitmann et al., 2020) performed on one of the 150 plots. SCALEMIC aimed to study the spatiotemporal variation of biodiversity at a local plot scale. A plot of 10 × 10 meter in the Swabian Alb, Germany, was divided into 60 subplots. At each of six dates from April to November 2011 all 60 subplots were sampled to analyze soil variables and microbial and plant diversity (Regan et al., 2014; Klaus et al., 2016; Stempfhuber et al., 2016; Richter-Heitmann et al., 2020).

### Determination of Biological and Chemical Variables

Soil water content was quantified by weighing a field fresh sample, drying for 3 days at 105°C, and then re-weighing. For the determination of pH, 10 g of sieved and air-dried soil were diluted (1:2.5 w:v) with 25 ml of 10 mM CaCl_2_ solution. pH was measured two times per sample with a glass electrode (WTW GmbH, Weilheim, Germany). Dry combustion with an elemental analyzer was used to determine total nitrogen (N_tot_), inorganic carbon (C_i_) and total carbon (C_t_) (VarioMax CN analyzer, Elementar Analysesysteme GmbH, Hanau, Germany) after samples had been ground and homogenized in a ball mill (RETSCH MM200, Retsch, Haan, Germany). Organic carbon content (C_org_) was calculated as C_org_ = C_t_–C_i_. Root biomass was quantified after cleaning the roots with distilled water and drying at 40°C (Solly et al., 2015). Nitrate (NO_3_^–^) and ammonium (NH_4_^+^) were extracted with 0.5 M K_2_SO_4_ and concentrations in the supernatant determined with an autoanalyzer (Bran & Luebbe, Norderstedt, Germany). The sum of NO_3_^–^ and NH_4_^+^ were considered as extractable mineral forms of nitrogen (N_mmin_). Soil microbial biomass carbon (C_mic_) and nitrogen (N_mic_) were determined in 10 g (fresh weight) samples by the chloroform-fumigation-extraction method (Vance et al., 1987). After fumigation with ethanol-free chloroform, samples were extracted with 0.5 M K_2_SO_4_ and extractable organic C (EC) and N (EN) were measured in supernatants with a Multi N/C 2100 (Analytik Jena, Jena, Germany). C_mic_ and N_mic_ were calculated from the differences in EC and EN-values of fumigated and non-fumigated controls.

Microbial phosphorus (P_mic_) was measured using a combination of methods (McLaughlin et al., 1986; Kouno et al., 1995). Three aliquots per sample of moist soil (corresponding to 2 g of dry soil) were weighed into 50 ml polyethylene tubes and 30 ml deionized water (H_2_Odeion) was added. A 0.5 M NaHCO_3_ conditioned resin strip (2 × 6 cm) was added to each samples. Subsequently, samples were shaken horizontally for 16 h with in either H_2_Odeion, H_2_Odeion plus 1 ml hexanol, or H_2_Odeion plus an internal phosphorus standard. The latter was added to correct for P release during fumigation. After shaking the resin stripes were removed and rinsed with H_2_Odeion to remove adhering soil. After addition of 30 ml NaCl/HCl, samples were shaken again for 2 h to desorb P. Phosphorus concentrations were measured with a continuous flow analyzer (CFA, AA3, XY2, Seal-Analytic, Norderstedt, Germany) at λ = 712 nm using the molybdate blue method (Murphy and Riley, 1962). A sorption curve between non-fumigated (H_2_Odeion) and P spiked samples was calculated to account for P sorption released during fumigation, and hexanol P (termed as P_mic_; Oberson and Joner, 2005; Bünemann et al., 2008) was computed.

For the determination of soil texture, soil organic matter was removed by oxidation with hydrogen peroxide and soil aggregates were dispersed. Afterward sand (2–0.063 mm), silt (0.063–0.002 mm), and clay (<0.002 mm) fractions were determined by sieving and sedimentation (DIN-ISO 11277).

For the determination of abundances of Lumbricidae, Diplopoda, Symphyla, and Pauropoda, one intact soil core per was sampled and hand sorted from all 150 sites (20 cm diameter and 10 cm deep cylindrical soil cores) between April 11 and 20, 2011.

Land-use intensity was investigated by interviewing land owners. With the variables of mowing frequency (m, number of cuts), grazing intensity (g, in livestock units day^–1^ ha^–1^ year^–1^) and fertilization level (f, in kg N ha^–1^ year^–1^) a dimensionless land-use intensity index (LUI) was calculated for each plot by dividing the mowing, grazing and fertilization components by the corresponding overall means and taking the square root of the sum of the three components (Blüthgen et al., 2012). The determination of plant root associated variables (biomass, chemical composition) was performed as described previously (Solly et al., 2014). Species richness of vascular plants, productivity and cover of vascular plants, herbs, legumes, grasses, bryophytes, litter and bare soil were calculated as reported before (Socher et al., 2012). For plant sampling per each of the 150 sites, we estimated the cover of all vascular plant species on 4 m × 4 m from mid-May to mid-June 2011 simultaneously in all regions and calculated the Shannon and Evenness diversity indices. Cover sums of the functional groups grasses, legumes and herbs were calculated by summing up the cover of all species in the respective groups. Furthermore, aboveground biomass was harvested in four randomly placed quadrats of 0.25 m^2^ adjacent to the vegetation record. Samples were dried and milled. After drying at 80°C for 48 h and weighing, an arithmetic mean of biomass per m^2^ for each plot was obtained as a measure of grassland productivity. To estimate nitrogen (N), carbon (C) phosphorus (P), potassium (K), calcium (Ca), and magnesium (Mg) concentrations biomass samples were analyzed by near-infrared reflectance (NIR) spectroscopy. We recorded the reflectance spectrum of each sample between 1,250 and 2,350 nm at 1 nm intervals. Each sample scan consisted of 24 single measurements, which were averaged to one spectrum. Accuracy of model predictions was checked by applying an external validation process. For further methodological details (see Kleinebecker et al., 2011).

### Extraction and Purification of RNA

RNA was extracted using the protocol of Lueders et al. (2004) that had been further adapted (Wüst et al., 2016). Briefly, soil samples were thawed on ice, 0.6 g were transferred into a 2 ml screw cap tube, 0.7 g of sterilized zirconium/silica beads (diameter, 0.1 mm), 750 μl sodium phosphate solution and 250 μl TNS-Buffer (Tris-HCl, sodium chloride, SDS) were added, and cells disrupted by beat-beating (6.5 m⋅s^–1^ for 45 s). After centrifugation, samples were extracted with phenol-chloroform-isoamyl alcohol (25:24:1) and chloroform-isoamyl alcohol (24:1) and nucleic acids pelleted by the addition of polyethylene glycol and centrifugation. Pellets were washed with ice cold 70% (v/v) ethanol, resuspended in 50 μl Tris-HCl buffer (pH 8.5), and the quantity and quality of the coextracts were checked by UV/Vis spectroscopy (NanoDrop ND-1000, Peqlab Biotechnologie, Erlangen, Germany).

Genomic DNA was removed by enzymatic digestion with DNase I (RNase free; Thermo Fisher Scientific, Waltham, MA, United States) according to the instructions of the manufacturer. RNA was precipitated with sodium acetate/isopropanol for 60 min on ice, washed with 70% ethanol and redissolved in RNase free water. RNA was quantified with RiboGreen dye (Quant-iT™ Ribo Green^®^ RNA Reagent and Kit, Life Technologies, Carlsbad, CA, U.S., microplate reader Tecan Infinite^®^ 200 PRO Tecan Group AG, Männedorf, Switzerland). Samples were stored at –80°C after addition of RNA RNase inhibitor (1 U⋅μl-1; RiboLock, Fermentas).

### Complementary DNA Synthesis and Amplification of 16S rRNA Genes

RNA was transcribed into cDNA using the GoScript™ Reverse Transcription System (Promega, Madison, WI, United States). Amplification of the variable V3 region was carried out with 20 μl cDNA mix as template using modified primers 341F and 515R (Muyzer et al., 1993; Bartram et al., 2011). These primers contain flow cell adapters and are complementary to the Illumina sequencing primers. The reverse primer also contained a 6-bp index allowing multiplexing of up to 97 samples per lane. All samples were amplified in duplicate. The reaction mix (final volume of 50 μl) contained 2 μM of each primer, 0.2 mM of each dNTP, 3% (v/v) DMSO and 0.02 U⋅ml^–1^ Phusion Taq polymerase (New England Biolabs^®^ Inc., Ipswich, MA, United States). Amplification proceeded by an initial denaturation step at 94°C for 5 min, followed by 15 cycles of 94°C for 15 s, 59°C for 15 s, 72°C for 15 s, and final extension step at 72°C for 7 min. The PCR products were purified on a 2% MetaPhor™ Agarose gel (Lonza group, Basel, Switzerland) and PCR products of the correct size (300–330 bp) were recovered from excised gel bands with the Nucleo Spin^®^ Gel and PCR Clean-up Kit (Macherey-Nagel, Düren, Germany).

### Sequencing, Analysis, and Matching of V3 16S rRNA Gene Amplicon Reads to Reference Operational Taxonomic Units From Clustered Full-Length 16S rRNA Gene Sequences

DNA concentrations were quantified with the Qubit^®^ dsDNA Assay Kit (Life Technologies, Carlsbad, CA, United States). Quality was checked with a 2100 Bioanalyser (Agilent Technologies, Santa Clara, CA, United States). Samples were sequenced in paired-end mode on individual lanes, with 50 samples per lane, of a HiSeq2500 and Illumina GAII (Illumina^®^, San Diego, CA, United States), yielding reads of 100 and 150 bp, respectively. Per lane an equivalent amount of genomic bacteriophage PhiX DNA was added to ensure sufficient cluster diversity. Reads were assigned to individual samples using the 6-bp index. Forward and reverse reads were trimmed to 100 bp and dimers as well as sequencing adapters were filtered out based on detection methods implemented in FastQC.^2^ Reads were joined using fastq-join (Aronesty, 2013) allowing 20% mismatch and a minimum overlap of six nucleotides. Converted FASTA files were checked for chimeras by Uchime (Usearch 5.2.32, Edgar et al., 2011) applying the GOLD database from ChimeraSlayer^3^ as a reference. Taxonomic-dependent analysis was performed using RDP Multiclassifier 1.1, which is essentially based on RDP classifer (Wang et al., 2007; Cole et al., 2009). A confidence value of 0.5 was applied for short read amplicon data. Based on the assignments of RDP Classifier retrieved by the –assign_outfile option, 25,616,775 of the joined reads were assigned to the phylum *Acidobacteria*.

All high quality full-length acidobacterial sequences (*N* = 13,087) available in SILVA release 119 (Quast et al., 2013) were downloaded (SILVA_119_SSURef_Nr99_tax_silva_trunc.fasta.gz) and augmented with 1,997 new sequences that had been generated through other in house projects. After de-replication a dataset of 12,231 unique 16S rRNA gene sequences was obtained. Sequences were aligned with Infernal 1.1 (-notrunc -g -matchonly) against its bacterial SSU model (Nawrocki and Eddy, 2013). The alignment was clustered with HPC-CLUST 1.1 (-t 0.9 -al true -dfunc nogap) (Matias Rodrigues and von Mering, 2014) which generated 5,450 reference OTUs at an identity cutoff of 97%. All 25,616,775 acidobacterial short read sequences obtained in the present study were then compared to the de-replicated reference sequences by BLAST (Altschul et al., 1997) employing a 99% identity cutoff (i.e., the maximum error rate of the Illumina sequencing approach). Subsequently, query sequences were assigned to full-length sequence reference OTUs using a fractionated approach. In case a read matched to several OTUs, each OTU got only a read value equal to one divided by the amount of matching OTUs (e.g., if one read match to 4 OTUs, each OTU got 0.25 read values).

### Phylogenetic Reconstruction and Generation of Ultrametric Trees

The dataset of full-length 16S rRNA sequences containing a representative sequence for each of the 5,450 OTUs were subjected to (ultrametric) phylogenetic reconstructions. Phylogenetic trees under the maximum-likelihood (ML) criterion were inferred with ExaML (Stamatakis and Aberer, 2013) version 3.0.7 under the GTR-CAT model by applying rapid hill-climbing starting from a maximum parsimony tree inferred with RAxML (Stamatakis et al., 2008) version 7.2.8. The tree was made ultrametric using LSD (To et al., 2016) version 0.1b after applying midpoint rooting as implemented in PAUP* (Swofford, 2002). Subdivision affiliation of sequences information was retrieved from the SILVA release 119 database.

### Determination of the Optimum Niche Value for Each Operational Taxonomic Unit for 41 Environmental Variables

**FIGURE 1:**
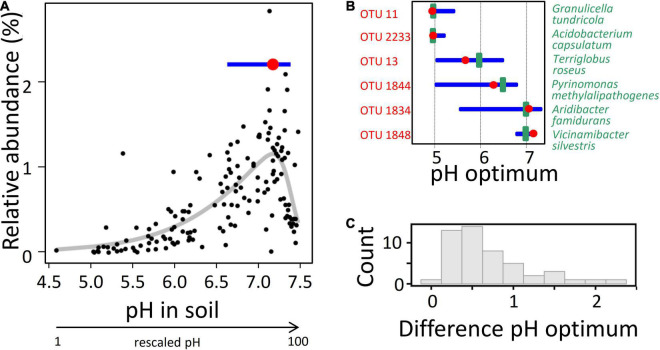
Determination of the interdependence between soil pH and the relative abundance of 16S rRNA sequence reads for *Acidobacteria* OTUs in Exploratory soils and comparison to the optima of corresponding laboratory strains. **(A)** Determination of the optimum pH for a single OTU using values from the 150 soil samples. Red point indicates the optimum response (at a soil pH of 7.2, which corresponds to a rescaled pH value of 90), blue horizontal line indicates the width of the inner ecological niche, as determined by employing the optimum response model with best fit (the thick gray line represents the best model fit; see section “Materials and Methods”). **(B)** Exemplary comparison of optimum pH (red point) and inner ecological niche width (blue horizontal line) of different OTUs (labeled in red) to the pH optimum for the growth of cultured representatives (green vertical line) belonging to the same OTU, as determined in the laboratory. **(C)** Histogram of the absolute deviation values between pH optima determined in cultivated representatives and the pH optima as determined from the optimum niche modeling for the corresponding OTU in the soils. All 49 described species for which pH optima were available were included. The results indicate that for the majority of strains the deviation of the two values is less than 0.6 pH unit (median = 0.51).

An initial list of 62 environmental variables was reduced to a final list of 41 variables in order to remove ecologically redundant and tightly correlated variables, retain putatively relevant variables and to minimize collinearity between variables. The mean of the absolute values of 820 pairwise Pearson correlation values was low (*r* = 0.19). Only few pairwise correlations (*N* = 14; 1.7%) were above a value of 0.7 but the variables were retained as they typically represent different ecological effects, such as soil moisture and carbon content. In any case, occasional collinearity is not critical here as these data are not used for regression approaches in which collinearity would result in variance inflation. The eHOF R package (Version 1.5.4) (Jansen and Oksanen, 2013) was then used to determine the specific value along the gradient of an environmental variable where the relative abundance of amplicon sequence reads of an OTU reached its maximum (i.e., the optimum niche value). The eHOF approach selects the best-fit out of the pre-determined model types (seven types of hierarchical logistic regression models, also known as Huisman–Olff–Fresco, HOF, models) for each OTU, using Akaike information criterion (AIC) and bootstrapping (here we used bootstrap = 50) to stabilize the model choice. In order to ensure comparability across environmental variable values, which are at different scales (Table 1), all environmental variables were normalized to a range from 1 to 100. The five tested models are of increasing complexity. The most important and simplest model type is number I, a flat response, that means there is no significant trend along the gradient for a particular OTU, hence there is no abundance optimum of the OTU throughout the ecological gradient. It is the null hypothesis and ensures that only OTUs with a clear response will be modeled with one of the other model types. Shape II is monotone sigmoid with a top at one end of the gradient, III is monotone sigmoid with a plateau below the maximal upper abundance value. Model type IV is the canonical form of species response, a unimodal symmetric model, V is a unimodal skewed model. As an example, the best fitting model for the data presented in Figure 1A is the unimodel skewed model V. The extended bimodal models (VI and VII) were not tested. Only OTUs were analyzed which occurred in at least 25 out of the 150 sites (these amounted to a total of *N* = 4,154 OTUs). From the best fitting model the peak of abundance was extracted as the optimum niche value. No optimum niche value could be determined in those cases where the flat model (model I) was identified as the best model. The degree of ecological divergence between OTUs was determined as Euclidian distances based on the optimum niche values of all 41 environmental variables by using the function euc.dist < - function(x1, x2) sqrt(sum((x1 - x2) ^ 2)). As the optimum niche values of 1 and 100 represent the extremes of an environmental gradient, the maximum ecological distance theoretically possible across 41 environmental variables is 634 [euc.dist(rep(1, 41), rep(100, 41)].

**TABLE 1:** List of 41 environmental variables.

Environmental variable	Abbreviations	Min.	Max.	Units	Additional information
Biomass grassland productivity	BM_grassland_	3	436	g m^–2^	
Land use index (LUI)	LUI	0.6	3.4	-	Type of land use in 2011
pH	pH	4.6	7.5	in 0.01 M CaCl_2_	Measured in CaCl_2_
Inorganic carbon	C_i_	0	78.5	g kg^–1^ soil	
Organic carbon	C_o_	12.2	359.5	g kg^–1^ soil	
Carbon/Nitrogen ratio	C/N	9	14.6	-	
Fine roots biomass	BM_roots_F_	0.1	53.4	g cm^–3^	<2 mm diameter
Coarse Roots biomass	BM_roots_C_	0	6.8	g cm^–3^	>2 mm diameter
Plant nitrogen	PL_M_	1.2	3.6	%	Concentration in aboveground biomass without litter
Plant carbon	PL_C_	42.7	46.1	%	Concentration in aboveground biomass without litter
Plant phosphorus	PL_P_	0.1	0.4	%	Concentration in aboveground biomass without litter
Plant potassium	PL_K_	0.6	3.8	%	Concentration in aboveground biomass without litter
Plant calcium	PL_Ca_	0.2	1.4	%	Concentration in aboveground biomass without litter
Plant magnesium	PL_g_	0.1	0.5	%	Concentration in aboveground biomass without litter
Sand	Sand	0.8	84.6	g kg^–1^ soil	0.063–2 mm
Silt	Silt	7.2	86.8	g kg^–1^ soil	0.002–0.063 mm
Clay	Clay	4.1	70.8	g kg^–1^ soil	<0.002 mm
Ammonium	NH_4_^+^	2.3	51.7	μg NH_4_^+^-N g^–1^	
Nitrate	NO_3_^–^	0	68.8	μg NO_3_-N g^–1^	
Mineral nitrogen	N_in_	4.4	112.3	μg N g^–1^	Sum of ammonium and nitrate
Carbon/nitrogen ratio in roots	Roots_C/N_	17.8	98.1	-	
Total carbon in roots	Roots_C_	28.8	48.5	%	
Total nitrogen in roots	Roots_M_	0.5	2.4	%	
pauropod abundance	Paur	0	16,270	ind m^–2^	
Earthworm abundance	Lumb	0	1,019	ind m^–2^	
Millipede abundance	Dipl	0	207	ind m^–2^	
Soil moisture	Soil_*H*20_	3.2	208.5	%	
Microbial biomass carbon	C_mic_	116	1,521	μg C g^–1^ soil dw	Microbial biomass carbon
Microbial phosphorus	P_mic_	3.5	81.9	mg kg^–1^	Hexanol fumigated
Ratio of microbial biomass carbon/microbial biomass nitrogen	C_mic_/N_mic_	4.8	10.4	-	
Shannon diversity index	Shannon	0.9	3.2	-	Plant Shannon diversity index
Evenness diversity index	Evenness	0.4	0.9	-	Plant evenness diversity index
Cumulative cover of all legumes (shrubs and herbs)	COV_legumes_incl_herb_and_shrubs_	0	60.5	%	Cumulative cover of all legumes (shrubs and herbs)
Species number of all legumes including legumes shrubs	NR_vasc_pl_	36.5	244.7	%	Cumulative cover of all vascular plants
Species number of all vascular plants	NR_vasc_pl_	12	64		Species number of all vascular plants
Cover of all bryophytes (view from above)	COV_bryoph_	0	90	%	Cover of all bryophytes (view from above)
Cover of litter (view from above)	COV_litter_	0	97	%	Cover of litter (view from above)
Cover of bare soil (view from above)	COV_bare_soil_	0	40	%	Cover of bare soil (view from above)

### Network Based Clustering and Multivariate Ordinations Based on Optimum Niche Values

Network based clustering using the multi-level modularity optimization algorithm for finding community structure (Blondel et al., 2008) as implemented in the R package igraph (Csardi and Nepusz, 2006) served to identify groups (network groups) of OTUs with similar optimum niche values (hence similar habitat adaptation) by means of correlation analysis. Pairwise Spearman rank correlation between all 4,154 OTUs was performed using numerical vectors comprising all 41 optimum response values. We explored a set of different rho values (0.4, 0.5, 0.6, 0.7, 0.725, 0.75, 0.775, 0.8, 0.825, 0.85, 0.875, 0.9, 0.925, 0.95, 0.98, 0.99, 0.8875, 0.96, 0.9375, 0.9125, 0.975) in order to identify how many network groups were detected at a specific correlation strength and how many OTUs were affiliated to any network group. At rho = 0.4, four network groups were identified which included all OTUs. Rho values of 0.9 and 0.925 yielded the largest number of network groups (*N* = 358 and 383, respectively), which even declined when rho was further increased. Moreover, such large rho values encompass only approximately 25% of all OTUs, with 75% of OTUs being discarded. Therefore, a rho value of 0.85 was taken as cutoff for detecting groups of ecologically similar OTUs, as 0.85 yields a reasonably large number of network groups (*N* = 264) encompassing still approx. 67% of all OTUs (*N* = 2,784). Hence, a rho value of 0.85 is regarded to be a reasonable compromise between gaining sufficient resolution and avoiding noise. The network was visualized using the GGally package with kamadakawai plotting mode. Multivariate ordinations (PCA and CA) based on the matrix of 4,154 OTUs and 41 optimum niche values were performed using the vegan package (Oksanen et al., 2018). The plot3D visualization of the first three PCA axes was done using the R package (version 1.1) (Oksanen et al., 2018). The mov-file showing a rotating 3D animation of the first three PCA axes (“PCA_rotate.mov”) was build using the animation R package (version 2.4) (Xie, 2013).

### Determination of Phylogenetic Signal of Optimum Niche Values

In order to determine to what extent phylogeny predicts the ecological similarity of OTUs, we quantitatively determined the phylogenetic strength of optimum niche values for each environmental variable by using Blomberg’s K statistic (Blomberg and Garland, 2002; Blomberg et al., 2003) and the area under the phylogenetic signal-representation (PSR) curve (PSR) as obtained from phylogenetic eigenvector regression (PVR) (Diniz-Filho et al., 1998, 2012a,b). The strength of phylogenetic signal describes the tendency (pattern) for evolutionary related organisms to resemble each other. The Blomberg’s K statistic estimates whether relatives resemble each other less (*K* < 1) or more (*K* > 1) than expected under Brownian motion evolutionary model. Overall, the larger Blomberg’s K statistic, the higher the phylogenetic strength of a specific trait. PVR starts by extracting eigenvectors [using a principal coordinate analysis (PCoA)] from pairwise distance matrices that describe the phylogenetic relationships among species and then use some of the eigenvectors (which can be selected using different criteria) to model trait variation with a standard ordinary least-squares (OLS) regression. The coefficient of determination (*R*^2^) of the multiple regression model is an estimate of phylogenetic signal. When sequential PVR models are computed by successively adding eigenvectors to model trait variation, it is possible to plot *R*^2^ against the accumulated eigenvalues associated with these eigenvectors and which generates a plot called a PSR curve. Negative values for the PSR curve (PSR area) indicate that a trait evolves faster than expected under Brownian motion. For both methods ultrametric phylogenetic trees were used as input, along with a numerical vector of the values of the environmental variable to be tested. Blomberg’s K statistic values were obtained using the function phylosignal() in the picante R package (Version 1.6-2) (Kembel et al., 2010). Phylogenetic eigenvector regression was performed using the PVR R package (Santos et al., 2012) (Version 0.2.1).

### Multivariate Ordination of Operational Taxonomic Units Corresponding to Cultivated *Acidobacteria* Species Based on Five Different Trait Matrices

The OTUs corresponding to cultivated *Acidobacteria* species (*N* = 63, including two Candidatus species “*Candidatus* Koribacter versatilis” and “*Candidatus* Solibacter usitatus”) were identified by > 99% similarity of the amplicon sequence to the reference full-length 16S rRNA gene sequence, yielding for 53 cultivated species a corresponding grassland OTU. At 99% similarity we did not find corresponding OTUs for nine species: *Acidicapsa acidisoli*, *Acidipila dinghuensis*, *Edaphobacter acidisoli*, *Edaphobacter bradus*, *Edaphobacter flagellates*, *Geothrix fermentans*, *Granulicella cerasi*, *Granulicella sibirica*, and *Holophaga foetida*. Six further species (*Bryobacter aggregatus*, *Telmatobacter bradus*, *Terriglobus aquaticus*, *Brevitalea deliciosa*, *Brevitalea cellulosilytica*, *Bryocella elongata*) were removed because optimum niche values could not be determined for 4–34 environmental variables. For 17 species optimum niche values were not available for one to two of the environmental variables (flat response model). In order to not to lose these species in a multivariate ordination we imputed the missing information by using the mean optimum niche value of the respective environmental variable (Legendre and Legendre, 2012), which we regard as a tolerable procedure as it affects less than 5% of the environmental variables. For 30 species a full set of 41 optimum niche values were available. Thereby, corresponding grassland OTUs were identified for 47 acidobacterial species with a reasonably complete set of 41 environmental optimum niche values. For the type strains of 45 species (the *Candidatus* species were omitted due to lack of data), physiological traits were retrieved from the taxonomic literature and the curated data Bac*Dive* database (Reimer et al., 2019) on ApiZym tests. Growth tests of carbon sources were grouped into (a) sugar and sugar derivatives, (b) amino acids, (c) metabolic intermediates, e.g., from the citric acid cycle, (d) proteins, (e) aromatic compounds and (f) polysaccharides. As by the nature of taxonomic descriptions this matrix in parts is highly uncomplete, we optimized in an interactive process the matrix to reach a good compromise by minimizing sparsity of the matrix while keeping as many traits and species as possible. This retained the datasets (a) and (c). For further numerical processing, the matrix was coded as 1 = negative, 2 = weak, and 3 = positive utilization. Additionally we compiled from the original species descriptions a numerical matrix on complex physiological growth traits: optimum pH for growth, optimum temperature for growth, maximum NaCl tolerance of growth, and relationship to oxygen (aerobic = 3, microaerophilic = 2, anaerobic = 1). The mean value was taken for those cases where a range of optimum pH and temperature values were provided. All downstream analyses were performed using the vegan R package. For each of the matrices we calculated Bray-Curtis dissimilarity values for each pair of strains while individually omitting those traits for which data availability was incomplete. The resulting distance matrices were subjected to an NMDS ordination analysis with 6 dimensions. The resulting score matrix was used for (a) producing the ordination plots and (b) performing a pairwise similarity analysis of the ordinations using a Procrustes analyses (function *protest* in vegan with 999 permutations). For each pairwise comparison the *protest* analysis was repeated 100 times in order to ensure stability of the significance value (mean of 100 repetitions).

### Other Statistical Methods

The R package ggplot2 (Wickham, 2016) was used for graphics. Multiple comparisons of means of groups by Null hypothesis significance testing (two-sided) were performed using the multcomp R package (Hothorn et al., 2008) as described previously (Herberich et al., 2010). The tested linear models were without interaction: lm(ecological divergence ∼ 16S rRNA gene dissimilarity + subdivision) and with interaction: lm(ecological divergence ∼ 16S rRNA gene dissimilarity * subdivision). The simple model without statistical interaction between 16S rRNA gene sequence divergence and subdivision does not allow for different rates of changes in ecological divergence with change in 16S rRNA gene sequence divergence across subdivisions, whereas the more complex model with interaction allows for different rates of changes across subdivisions. Both models were compared via ANOVA and the AIC and in case both models yield similar results in ANOVA and AIC, the more simple model should be further investigated. Model validation was performed as following: The residual plots against both 16S rRNA gene dissimilarity and subdivisions indicated homogeneity. A Q-Q plot indicated a good fit between standardized residuals and the theoretical quantiles.

The currently known counts of different *Acidobacteria* full-length 16S rRNA gene sequences was determined by analyzing the SILVA 138.1 (SILVA_138.1_SSURef_NR99_tax_silva_trunc.fasta.gz), assessed on 31st of March 2021, supplemented with the 1,997 in-house sequences. The “isolation source” metadata tags in SILVA 138 was analyzed to determine the proportion of counts affiliated to either soil or (semi-) aquatic sediments.

## Results and Discussion

### Optimum Niche Values of Soil *Acidobacteria* Across Multidimensional Environmental Gradients

A total of 225 million bacterial reads were obtained across the 150 soil samples, with an average of 1.5 million per sample, from which 25,616,775 acidobacterial reads were retrieved. Their fraction constituted between 6.9 and 19.4% and on average 12.6% across the 150 soil samples. Alpha-rarefaction curves indicated a saturating sampling depth across the three exploratory regions (data not shown). The acidobacterial reads were assigned to a backbone phylogenetic tree of full-length 16S rRNA genes sequences constructed for 5,450 reference OTUs (see section “Materials and Methods”) using a 99% identity cutoff, which made it possible to compute the relative abundances of 16S RNA gene transcripts for the different *Acidobacteria* OTUs in the different soil samples. The advantage of 16S rRNA gene sequences over metagenomic sequence data is to trace distinct OTUs across multiple samples. 4,154 of the reference OTUs were sufficiently covered by 16S rRNA transcripts (i.e., detected in > 25 of the plots) to allow determination of the optimal niche via Huisman–Olff–Fresco (HOF) models, for each OTU and for each of the 41 environmental variables measured. The latter comprised diverse physicochemical soil properties, microbial biomass variables, and a multitude of variables for plants and soil animals, all of which may affect microbial activity (Wardle et al., 2004). The 150 Biodiversity Exploratory soils chosen covered broad gradient ranges of these environmental variables (Table 1). So far, the HOF modeling approach (Jansen and Oksanen, 2013), which uses hierarchical logistic regressions to find niche optima along environmental gradients, has primarily been applied to eukaryotes. Only recently this approach has successfully also been applied to soil bacteria (Tripathi et al., 2018; Jones et al., 2021), although soil heterogeneity at the microscale level (micrometer) cannot be taken into account. To test our approach, we compared the optimum niche value predicted for pH for individual OTUs with the corresponding pH growth optimum determined in laboratory cultures of isolates from the same OTU. Notably, the pH optima of cultivated representatives were largely commensurate with those determined for their respective OTU in the Exploratory soils (Figure 1), which confirms the validity of our culture-independent approach toward the analysis of habitat adaptation of *Acidobacteria*.

### High Ecological Diversity and Its Evolution in Different *Acidobacteria* Subdivisions

**FIGURE 2:**
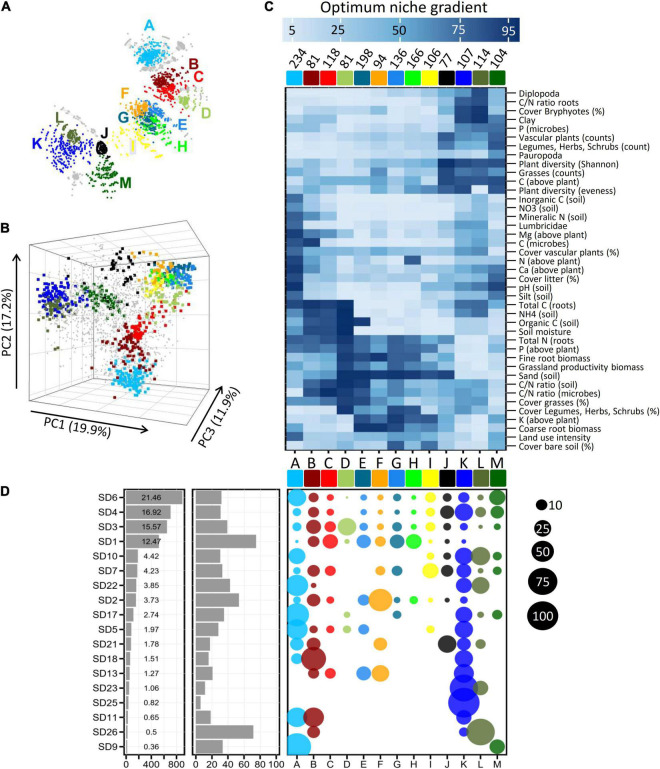
Groups of OTUs (A–K, colored) with similar ecological adaptations (niches) inferred from network based clustering **(A)** and Principal Component Analysis **(B)**. Small gray dots in **(B)** denote the position of OTUs which are not included in any of the major ecological groups (see also the full network in Supplementary Figure 1). **(C)** Clustered heatmap on mean optimum response values from OTUs belonging to the 13 major ecological groups. Values at the top of the heatmap give the counts of OTUs per ecological group. The bluish color gradient on top indicates the optimum niche values on a scaled gradient (1–100) of the environmental variables (Table 1). **(D)** Left panel: Counts of OTUs per subdivision (SD). Values in the panel represent proportional contribution (%). Middle panel: Proportions of OTUs per subdivision which are affiliated to the 13 ecological groups. Right panel: Proportions of OTUs across the 13 ecological groups. Sizes of the bubbles reflect the percentages as indicated on the far right.

In order to identify ecologically similar OTUs, a correlation matrix based on pairwise correlations of optimum niche values between the different OTUs was analyzed using network based clustering. A substantial fraction of 1,616 (38.8%) OTUs formed 13 major network groups (Figure 2A and Supplementary Figure 1) with each group consisting of ecologically highly similar OTUs. These clusters were further supported by a Principal Component Analysis (Figure 2B and Supplementary File “PCA_rotate.mov”) and also Correspondence Analysis (not shown). The remaining OTUs (61.2%) did not exhibit sufficiently similar ecological patterns and therefore did not form additional network groups of any considerable size. In their entirety, the OTUs that were detected in the active fraction of *Acidobacteria* cover a wide range of possible combinations of ecological preferences (i.e., the gray points cover most of the three-dimensional space in Figure 2B), whereas one third of the OTUs occupy only a limited number of discrete ecological niches (colored points in Figures 2A,B) that are characterized by distinct combinations of the different environmental variables (Figure 2C).

Among the 4,154 OTUs analyzed in the grassland soils, members of *Acidobacteria* subdivisions 1, 3, 4, and 6 dominated (Figure 2D left panel). To date, habitat preferences of different *Acidobacteria* subdivisions have been studied for very few environmental factors, in particular pH, indicating that members of SD4 and SD6 thrive in soils with moderate pH, whereas members of SD1 and SD3 seem to prefer acidic soils (Jones et al., 2009). However, our multidimensional analysis across gradients of 41 environmental variables revealed that the 13 complex ecological niches that could be determined were neither congruent with, nor exclusive to, specific phylogenetic groups: SD6 and SD4 *Acidobacteria* predominantly fell into ecological groups A/M or J/K/M, respectively (Figure 2D, right panel) that show preferences for slightly acidic to neutral pH, but still have different pH optima (Figure 2C) and also prefer different combinations of the other 40 environmental variables. Conversely, the ecological groups B/C/E/G/H are dominant among SD1 *Acidobacteria* and prefer low pH values but some representatives of SD4 and SD6 also belong to these ecological groups (Figures 2C,D, right panel). Evidently, different OTUs of the same *Acidobacteria* subdivision occupy different ecological niches and as a result, individual subdivisions demonstrate a previously unknown broad ecological variability.

**FIGURE 3:**
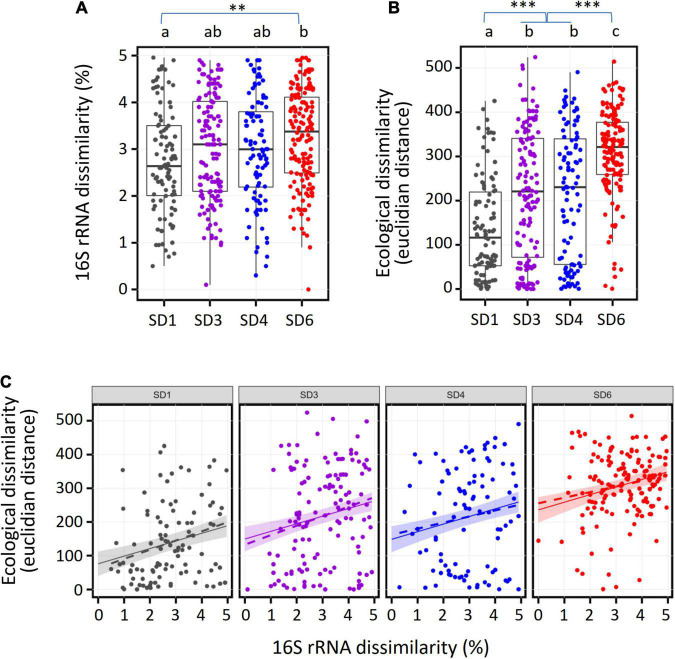
Comparison of the pairwise dissimilarity of full length 16S rRNA gene sequences with the corresponding ecological divergence for the major subdivisions of soil *Acidobacteria*. Each dot represents a pair of OTUs (SD1, *N* = 102; SD3, *N* = 132; SD4, *N* = 96; SD6, *N* = 152) which forms a unique terminal clade (i.e., a genus) at the tip of the phylogenetic tree of the 4,154 OTUs. **(A)** Distribution of 16S rRNA dissimilarity values, **(B)** Distribution of ecological dissimilarity values. The significance code for multiplicity adjusted *p*-values (multcomp test) is 0 “^***^”0.001 “^**^”0.01. **(C)** Scatterplot of 16S rRNA and ecological dissimilarity values per subdivision. Dotted lines reflect the linear regression line from the data. Solid lines and the shaded areas reflect the regression line and its 95% confidence intervals, respectively, as predicted from simulated 16S rRNA dissimilarity values (*N* = 50 per SD) using the best fitting linear model (without interaction of 16S rRNA dissimilarity with subdivisions).

Because of the high ecological diversity detected within single *Acidobacteria* subdivisions, we analyzed the extent to which ecological divergence is coupled to phylogenetic sequence divergence of 16S rRNA genes during speciation events in each subdivision. For each subdivision, pairs of OTUs that formed a terminal clade in the phylogenetic tree and showed ≤ 5% dissimilarity of their full length 16S sequences were chosen and the pairwise Euclidian distance across the optimum niche values of the 41 environmental variables between both OTUs was calculated. This OTU selection enabled the most sensitive analysis of ecological differentiation by focusing on the most recent evolutionary divergence events during the speciation within single acidobacterial genera. Overall, the four subdivisions showed a similar distribution of 16S rRNA gene sequences dissimilarity values, although dissimilarity values between OTUs of SD1 were somewhat smaller than those of SD6 (Figure 3A). By comparison, the distributions of pairwise ecological similarities in the different subdivisions differed significantly (Figure 3B), suggesting that the ecological divergence during speciation events was systematically higher in SD6 than in SDs 3 and 4, and that ecological divergence was lowest in SD1.

Such systematic differences in the intragenus ecological distances of different SDs could be due to constantly differing rates of ecological divergence during the speciation events or by short bursts of rapid niche separation in only some of the SDs. In order to evaluate both possibilities, we fitted linear models with ecological divergence as response variable and 16S rRNA gene dissimilarity and subdivision affiliation as explanatory variables, without and with statistical interaction between 16S rRNA gene dissimilarity and subdivision affiliation, respectively. The ANOVA comparison of both models (*F*-test, DF = 3, *F* = 0.35, *p* = 0.78) did not show a significant difference between both models, suggesting the simpler model (without interaction) could be used. In support of this, the AIC value favors the model without interaction (AIC = 6,010) in comparison to the model with interaction (AIC = 6,015; models with lower AIC are to be preferred, models with difference in AIC of ≤ 2 are regarded as equally good). The model without interaction was statistically significant (*F*-statistic: 35.81 on 4 and 477 DF, *p*-value: < 2.2e-16) and identified a statistically significant (*p* < 0.0001) increase in ecological divergence of 22.6 per percent change in 16S rRNA gene sequence dissimilarity. The intercepts (value of ecological divergence when 16S rRNA gene dissimilarity is zero) are 76 for SD1, 150 and 149 for SD3 and SD4, respectively (both significantly larger than the value for SD1, *p* < 0.0001), and 236 for SD6 (significantly larger than SD1 and SD3/SD4 at *p* < 0.0001). Notably, the intragenus values of Euclidian ecological distance reached 524 (Figure 3B), which is close to the maximum Euclidian ecological distance of 634 that can be reached with 41 environmental variables (see section “Materials and Methods). This indicates that even *Acidobacteria* of the same genus can be ecologically highly diverse. Given these results, the initial niche separation at the onset of speciation is burstlike and is most pronounced in SD6 and lowest in SD1. This implies that the potential for initial ecological divergence and adaptation is the smallest in SD1 but the largest in SD6. Interestingly, this conclusion is commensurate with the genome sizes of the different SDs, which increase from 5.4 ± 1.3 (Mb, mean ± S.D.) in SD1 (45 genomes) to 7.8 ± 2.9 in SD3 (3 genomes) up to 9.4 ± 1.9 in SD6 (mean ± deviation from mean, 2 genomes; this includes author’s unpublished results). The only available genomes from SD4 belong to thermophilic strains from continental hydrothermal springs and are therefore excluded from this comparison. Recently, the evolutionary response of aquatic bacteria during the adaptation to new environments was found to depend on genome size among other factors (Scheuerl et al., 2020). Our findings indicate that the evolutionary changes in soil *Acidobacteria* are most pronounced at the onset of speciation and highest for members of SD6 in accordance with their larger genomes. Larger acidobacterial genomes have been shown to encompass larger numbers of paralogous genes that can originate from gene duplication, producing genes with novel functions or genes with similar function that are expressed under different growth conditions (Eichorst et al., 2018). Because of their size, larger genomes are also subject to higher numbers of mutations. Such an increased genetic potential of *Acidobacteria* with larger genomes likely is advantageous for their colonization of soils that feature fluctuating ecological conditions.

### Phylogenetic Evolution of Ecological Adaptations

**FIGURE 4:**
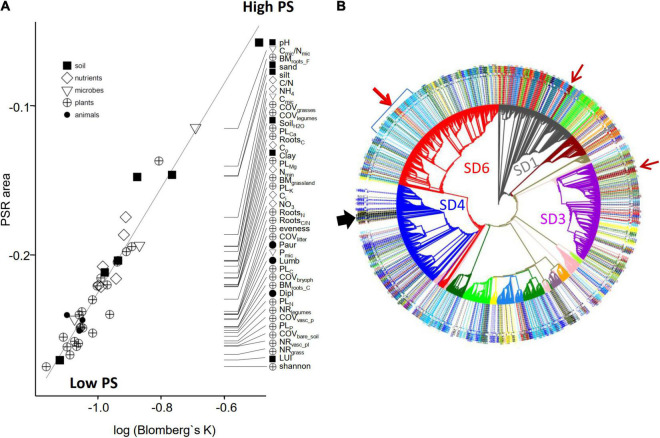
Comparison of ecological and phylogenetic similarity. **(A)** Phylogenetic strength (PS) estimated independently by Blomberg’s K and phylogenetic eigenvector regression. For all tested environmental variables, the strength of the phylogenetic signal was significant (Blomberg’s K always < 1; *p* < 0.001). By convention, the area under the phylogenetic-signal-representation curve (PSR area) is considered negative if the phylogenetic signal is smaller than expected under Brownian motion. Black line shows the regression line. **(B)** Maximum Likelihood tree of 4,154 full length 16S sequences. Subdivisions are colored. The color code of the outer ring indicates to which of the 13 ecological groups (Figure 2) the tips belong to, otherwise the tips are not colored. See “Results” section for further figure elements such as arrows.

More closely related species tend to be more similar to each other than expected by chance alone, creating a phylogenetic signal (Blomberg and Garland, 2002). The strength of the phylogenetic signal describes the tendency for evolutionary related organisms to resemble each other in their traits. For example, for a trait with a high phylogenetic signal the members of a specific phylogenetic clade are very similar with respect to this trait but quite different from the members of another clade, which themselves are again very similar among themselves. On the opposite, for a trait with low phylogenetic signal closely related organisms may be highly different in this trait whereas phylogenetically distantly related organisms may be highly similar with respect to this trait. In order to differentiate between individual environmental variables that had the strongest selective effects on the diversification of *Acidobacteria* during different periods of their evolution, we employed two different measures of the phylogenetic signal of traits, Blomberg’s K (Blomberg and Garland, 2002) and phylogenetic eigenvector regression PVR (Diniz-Filho et al., 1998, 2012a,^2015^; Figure 4A). The relationship of the phylogeny and the traits (here: ecological adaptations as deduced from optimum niche values) was analyzed based on the strength of phylogenetic signal for each of the 41 variables (Figure 4A). For each of the 41 variables, Blomberg’s K values ranged from 0.31 to 0.61 and were highly significant (*p* < 0.001) suggesting phylogenetic overdispersion and convergent evolution (see section “Materials and Methods). This is further supported by the negative PSR area values (Figure 4A, see section “Materials and Methods”). Therefore, the adaptations to specific values along the gradients of the environmental variables must have evolved multiple times during the evolution of *Acidobacteria*. However, despite the overall trend for overdispersion for all traits, there are distinctions in the strength between the 41 variables. For example, pH reveals the largest Blomberg’s K and PSR area values (Figure 4A) and therefore represents the environmental variables with the strongest phylogenetic signal. This supports the long-standing observation of an adaptation to different pH values and suggests that this adaptation represents an ancient phylogenetic signal which was relevant at the early evolutionary split of *Acidobacteria* into subdivisions. Throughout subsequent evolutionary history, adaptations to other soil physicochemical variables (texture, moisture) and to soil nutrients (C, N content) became relevant, as suggested by their weaker phylogenetic signal (Figure 4A). Based on our results, plant-related variables only became more important for the ecological adaptation of soil *Acidobacteria* in their recent evolutionary history (Figure 4A).

As already indicated by the phylogenetic overdispersion, the ecological preferences of OTUs often contrasted with their phylogenetic position based on full-length 16S rRNA gene trees (Figure 4B), indicating convergent evolution. Convergence occurred at different phylogenetic levels and was retained in clades of different phylogenetic depths. Thus, two small and phylogenetically shallow clades, one within SD1 and one in SD3 (brown arrows, Figure 4B) were found to belong to ecological group B (compare Figure 2C). An illustrative example of a phylogenetically deeper cladogenic adaptation is apparent for SD6. Whereas the majority of soil *Acidobacteria* of SD6 shows ecological preferences which are typical for groups J/K/L/M (see Figure 2D), a larger clade emerged within SD6 that encompasses a considerable number of OTUs that fall into ecological group A (blue bracket, Figure 4B). The ecological niche of group A is characterized by higher values for pH, organic C, inorganic nitrogen, soil moisture, plant root biomass and plant cover, litter cover, and earthworm abundance, which together are indicative of nutrient rich, active soils with the highest land use intensity (compare Figure 2C). Subsequently, a small but distinct subclade emerged within this clade, with adaptations characteristic of ecological group B and C (red/brown arrow, Figure 4B). This secondary evolutionary event occurred in adaptation to soils with even greater organic C and soil moisture values, but at the same time substantially lower pH values.

A second example of convergent evolution of a subclade can be observed in SD4. The OTUs of SD4 show a broad range of ecological adaptations (Figure 2D), and mostly fall into ecological groups M and K. However, a small but distinct clade emerged during the evolution of SD4 with lineages that mostly belonged to ecological group J (black arrow, Figure 4B), adapted to plots within the German grasslands that had sandy soils, lowest soil moisture and lowest nutrient content (organic C, N_min_, NO_3_, NH_4_), hence providing dry and oligotrophic conditions. Correspondingly, this clade also encompasses four *Acidobacteria*l isolates (*Aridibacter famidurans*, *Aridibacter kavangonensis*, *Aridibacter nitroreducens*, *Blastocatella fastidiosa*) which have been isolated from rather dry and nutrient habitats in African Sub-Saharan Kalahari soils (Foesel et al., 2013; Huber et al., 2014, 2017).

### Specific Niche Adaptation Also Explains Seasonal Changes in the Abundance of Active Operational Taxonomic Units

Based on the results described in the preceding sections, the distribution of active *Acidobacteria* across multiple environmental gradients in grasslands soils is consistent with distinct ecological adaptations of the different OTUs. Since soil environmental conditions in the temperate zone undergo pronounced seasonal changes, the composition of active soil bacteria may also change with time according to the availability of their ecological niches. We therefore tested the transferability of predictions of our niche modeling by analyzing the results of an independent, and seasonal, study (SCALEMIC) which had been conducted in parallel and in the same year as our study (2011) on a grassland plot in one of the Biodiversity Exploratories (Schwäbische Alb) (Richter-Heitmann et al., 2020). In several subplots of this grassland plot, bacterial community compositions were observed to change significantly within 6 weeks between May to June. Specifically, 32 *Acidobacteria*l OTUs, primarily from SD1 and SD2 (15, and 12 OTUs, respectively) were observed to transiently reach unusually high relative abundances (Richter-Heitmann et al., 2020) in June. Since the plant biomass in the same subplots also reached peak values in June, since the changes in *Acidobacteria*l community composition accompanied an increase in surface cover by the common grass *Dactylis glomerata* (Richter-Heitmann et al., 2020), and since certain members of SD1 may interact with plants (Kielak et al., 2016b), we hypothesized that the observed changes could be explained by the specific ecological adaptations of the OTUs that had increased in abundance. Indeed, the 32 OTUs exhibiting a dynamic response mostly fell into ecological groups C, D, E, F and H (4, 1, 6, 11, and 5 OTUs per group, respectively). Based on our optimum niche modeling, these very ecological groups were adapted to grassland soils with high root biomass and high grassland productivity. Therefore our niche modeling is in line with the independent seasonal observations on a selected plot in the Biodiversity Exploratories.

### How Do the Modeled Niches of Cultured *Acidobacteria* Compare to Their Metabolic Traits?

With our approach, the ecological niches of uncultured *Acidobacteria* in European temperate grasslands were defined through a quantitative analysis of niche optima values for active OTUs with respect to different soil geochemical parameters as well as characteristics of plants and soil meso- and macrofauna. Although cultivated isolates are available for only 63 *Acidobacteria*l species, 47 of these species (including the two *Candidatus* species) actually were representatives of OTUs that were analyzed in the present work. This provided the opportunity to compare the described metabolic characteristics of cultivated representatives to their niches that were inferred in the present investigation for the same OTU.

**FIGURE 5:**
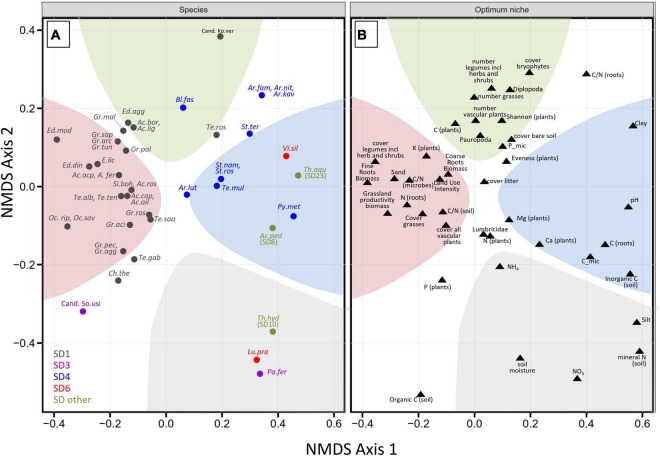
Correspondence of modeled niches with carbon substrate utilization. NMDS ordination plot on Bray-Curtis distance (*k* = 3, stress = 0.08, the non-metric fit between ordination distance and observed dissimilarity based on stress was *R*^2^ = 0.994) based on optimum niche values of the OTUs that corresponded to cultured isolates (type strains of validly named species and two *Candidatus* species). To enhance visualization, the NMDS scores from species and optimum niche values were separated into two panels **(A,B)**, respectively. The colored background areas in both panels reflect adaptations of the cultured species **(A)** to high values of the indicated environmental variables **(B)** (see text). *Ac.cap*, *Acidobacterium capsulatum*; Ac.ail, *Acidobacterium ailaaui*; Te.ros, *Terriglobus roseus*; Te.alb, *Terriglobus albidus*; Te.saa, *Terriglobus saanensis*; Te.ten, *Terriglobus tenax*; Gr.pal, *Granulicella paludicola*; Gr.pec, *Granulicella pectinivorans*; Gr.ros, *Granulicella rosea*; Gr.agg, *Granulicella aggregans*; Gr.aci, *Granulicella acidiphila*; Gr.arc, *Granulicella arctica*; Gr.mal, *Granulicella mallensis*; Gr.tun, *Granulicella tundricola*; Gr.sap *Granulicella, sapmiensis*; Ed.mod, *Edaphobacter modestus*; Ed.agg, *Edaphobacter aggregans*; Ed.lic, *Edaphobacter lichenicola*; Ed.din, *Edaphobacter dinghuensis*; Ac.bor, *Acidicapsa borealis*; Ac.lig, *Acidicapsa ligni*; Ac.fer, *Acidicapsa ferrireducens*; Ac.acp, *Acidicapsa acidiphila*; Ac.ros, *Acidipila rosea*; Oc.rip, *Occallatibacter riparius*; Oc.sav, *Occallatibacter savannae*; Te.gab, *Terracidiphilus gabretensis*; Si.boh, *Silvibacterium bohemicum*; Pa.fer, *Paludibaculum fermentans*; Bl.fas, *Blastocatella fastidiosa*; Ar.fam, *Aridibacter famidurans*; Ar.kav, *Aridibacter kavangonensis*; Ar.nit, *Aridibacter nitratireducens*; Te.mul, *Tellurimicrobium multivorans*; St.ter, *Stenotrophobacter terrae*; St.ros, *Stenotrophobacter roseus*; St.nam, *Stenotrophobacter namibiensis*; Ar.lut, *Arenimicrobium luteum*; Py.met, *Pyrinomonas methylaliphatogenes*; Ch.the, *Chloracidobacterium thermophilum*; Vi.sil, *Vicinamibacter silvestris*; Lu.pra, *Luteitalea pratensis*; Ac.ped, *Acanthopleuribacter pedis*; Th.hyd, *Thermotomaculum hydrothermale*; Th.aqu, *Thermoanaerobaculum aquaticum*; Cand. Ko.ver, *“Candidatus* Koribacter versatilis”; Cand. So.usi, *“Candidatus* Solibacter usitatus.”

NMDS ordination of the OTUs of these 47 species revealed clear differences in the combinations of optimum niche values for abiotic and biotic variables (Figure 5 and Supplementary Figure 2). The analysis suggests that many members of subdivision SD1 coincide with sandy, low-pH soils that have high plant cover, particularly of legumes, a high root biomass, and high plant productivity and land use intensity (red sector in Figure 5). While an adaptation of SD1 *Acidobacteria* to low pH values has previously been inferred from the abundance distribution of their 16S rRNA genes (Jones et al., 2009; Naether et al., 2012; Foesel et al., 2014), future investigations must show the extent to which these ecological preferences are driven by a direct interaction with plants, like the plant growth promoting effect on the non-legume *Arabidopsis thaliana* that was previously reported for members of the genus *Granulicella* and the type strain of *Acidicapsa ligni* (Kielak et al., 2016b). In contrast to most members of SD1, close relatives of *Pyrinomonas methylaliphatogenes* (SD4), *Vicinamibacter silvestris* (SD6), *Acanthipleuribacter pedis* (SD8), and *Thermoanaerobaculum aquaticum* (SD23) prefer clay-rich, carbonate-containing, more alkaline soils (blue sector in Figure 5), whereas, moist, N-rich, silty soils select for relatives of *Luteitalea pratensis* (SD6), *Paludibaculum fermentans* (SD3), and *Thermotomaculum hydrothermale* (SD10) (gray sector in Figure 5). Finally, our results suggest an adaptation of certain members of SD 1 (“*Candidatus* Koribacter versatilis,” *Terriglobus roseus*) and of SD4 (*Blastocatella fastidiosa*) species to nitrogen-poor, drier soils that sustain highly diverse plant communities (green sector in Figure 5). Notably, the two representatives each of SDs 3 and 6 occupied very different ecological niches based on our data analysis. The overall lack of a clear grouping of OTUs from the same SD in our analysis stresses again our earlier finding that ecological divergence among more closely related *Acidobacteria* of the same SD can be considerable (see also Figure 3) and hence occur more rapidly than the ecological divergence between some members of different SDs. We therefore caution against an assignment of particular *Acidobacteria* SDs to a specific ecological niche.

For comparison, physiological traits of the characterized 45 type strains were retrieved from their original taxonomic descriptions in the literature as well as the curated Bac*Dive* database (Reimer et al., 2019). However, these descriptions of type strains provided very different amounts of data. Therefore, strains that had information on less than 70% of the characteristics and those characteristics that had information for less than 50% of the strains were deleted in an iterative process (see section “Materials and Methods). The final datasets (Supplementary Figure 2) comprised 29 strains with sufficient information on (i) 19 different extracellular enzymes, (ii) the utilization of 26 sugars and sugar derivatives, (iii) the utilization of 17 metabolic intermediates as well as (iv) four complex physiological traits (optimum pH, temperature, NaCl content and oxygen demand). Finally, these four datasets for phenotypic characteristics of the 29 strains and the optimum niche values for the corresponding 29 OTUs were used to calculate pairwise similarity values which were then compared by NMDS ordination (Supplementary Figure 3). The type strains grouped differently depending on the traits analyzed, but the phenotypic similarity between members of SD1 and the dissimilarity between the two representatives of SD6 was particularly evident for the spectrum of sugar substrates utilized and for the extracellular enzymes (Supplementary Figure 3), respectively. Less distinct groupings were observed for the metabolic intermediates utilized.

Theoretically, the preferred association with plant roots, plant biomass and productivity of SD1 *Acidobacteria* as indicated by our analysis of optimum niche values (Figure 5) could be due to a preferred utilization of metabolic intermediates that dominate among root exudates (e.g., sugars, sugar alcohols, organic acids), and/or on their capacity to degrade plant-derived biomass through extracellular enzymes. For instance, the production of extracellular enzymes involved in the degradation of plant-derived biopolymers has been used to infer a role in organic matter decomposition in soils (Banerjee et al., 2016; Garcia-Fraile et al., 2016; Belova et al., 2018; Hausmann et al., 2018; de Chaves et al., 2019; Ivanova et al., 2020). We therefore tested the correlation between the ordination of optimum niche values and that of the four different phenotypic datasets by Procrustes rotation (Table 2). While the patterns of extracellular enzymes, of the utilization of sugars/sugar derivatives and of complex traits showed reasonably high and statistically significant correlations (*p* = 0.002–0.02), these patterns were not congruent with the distribution of the optimum niche values based on our observations of the real environmental distribution of *Acidobacteria*l OTUs. Patterns of utilization of metabolic intermediates showed weaker correlations with those of sugar utilization, but again not with the distribution of optimum niche values. This is in line with the recent finding that the chemical composition of root exudates has only a limited impact on the composition of rhizosphere bacterial communities compared to the properties of the surrounding soils (Vieira et al., 2020). Clearly, the standard procedures that are applied for routine phenotypic characterization of newly isolated bacteria yield only limited information on their realized niche in the natural environment. For example, the affinity and level of expression of extracellular enzymes under natural conditions might be more important than their mere presence or absence in a bacterium. We suggest that future characterizations consider such additional phenotypic traits of ecological relevance, as indicated by optimum niche value analyses such as the one described here. Based on our data, the determination of optimum niche values as developed in the present work can also provide first indications on the possible niche differentiation between more closely related *Acidobacteria* and hence provide additional information on potential drivers of bacterial speciation processes. In addition, optimum niche values provide valuable information for future cultivation trials of not-yet-cultivated types of *Acidobacteria*.

**TABLE 2:** Correlation and significance of trait matrices of 29 *Acidobacteria*l species in Procrustes comparisons.

	Optimum niche	Enzyme activity	Growth on sugars	Growth on metabolic intermediates
Enzyme activity	0.377 (0.271)			
Growth on sugars	0.348 (0.199)	**0.464 (0.006)**		
Growth on metabolic intermediates	0.323 (0.735)	0.351 (0.537)	**0.424 (0.019)**	
Complex growth traits	0.337 (0.295)	**0.466 (0.005)**	**0.544 (0.002)**	0.283 (0.759)

*Values represent correlation values in a symmetric Procrustes rotation and the statistical significance in brackets (Mean of independent 100 permutation runs; the null hypothesis is that matrices do not differ). Significantly similar matrices are in bold.*

## Data Availability Statement

The datasets presented in this study can be found in online repositories. The names of the repository/repositories and accession number(s) can be found below: https://www.ebi.ac.uk/ena, PRJEB12895; https://www.bexis.uni-jena.de/, 14326, 14346, 15286, 14686, 14446, 14448, 14447, 14567, 14766, 15766, 17046, 25086.

## Author Contributions

JO and JS conceived the study, analyzed the data, and wrote the manuscript with help from MF, EK, SM, YO, NH, and VW and the other authors. JR and CM developed HPC-CLUST. BB, MG, and SH processed the phylogenetic data. VB, KB, BF, MWF, VK, TK, RB, KH, TR-H, SM, DP, KR, BS, IS, MS, ESo, and ESc collected and processed the raw data. All authors contributed to the article and approved the submitted version.

## Conflict of Interest

The authors declare that the research was conducted in the absence of any commercial or financial relationships that could be construed as a potential conflict of interest. The reviewer IB declared a shared affiliation with one of the authors, NH, to the handling editor at the time of review.

## Publisher’s Note

All claims expressed in this article are solely those of the authors and do not necessarily represent those of their affiliated organizations, or those of the publisher, the editors and the reviewers. Any product that may be evaluated in this article, or claim that may be made by its manufacturer, is not guaranteed or endorsed by the publisher.

## Footnotes

1
http://www.biodiversity-exploratories.de

2
http://www.bioinformatics.babraham.ac.uk/projects/fastqc/

3
http://drive5.com/otupipe/gold.tz

## Supplementary Material

Supplementary Figure 1 Network graph of all 4,154 OTUs. Edges link nodes (OTUs) which have a Spearman correlation value > 0.85 across 41 optimum niche values. Color coding is according to the 13 network groups with the largest numbers of OTU.

Supplementary Figure 2 Phenotypic properties of 29 *Acidobacteria*l type strains.

Supplementary Figure 3 NMDS analysis of 29 *Acidobacteria*l type strains by 5 different trait matrices.

Supplementary Information 1 The MOV file PCA_rotate.mov shows a rotating 3D Principal Component Analysis ordination. Colors are the same as in Figure 2B. Text shows the axis scores for the environmental variables.
