# Supplementary figures and images for: The Evolution of Ecological Diversity in Acidobacteria
Source: Front Microbiol. 2022 Feb 2;13:715637. doi: 10.3389/fmicb.2022.715637 (PMC8847707; doi:10.3389/fmicb.2022.715637)

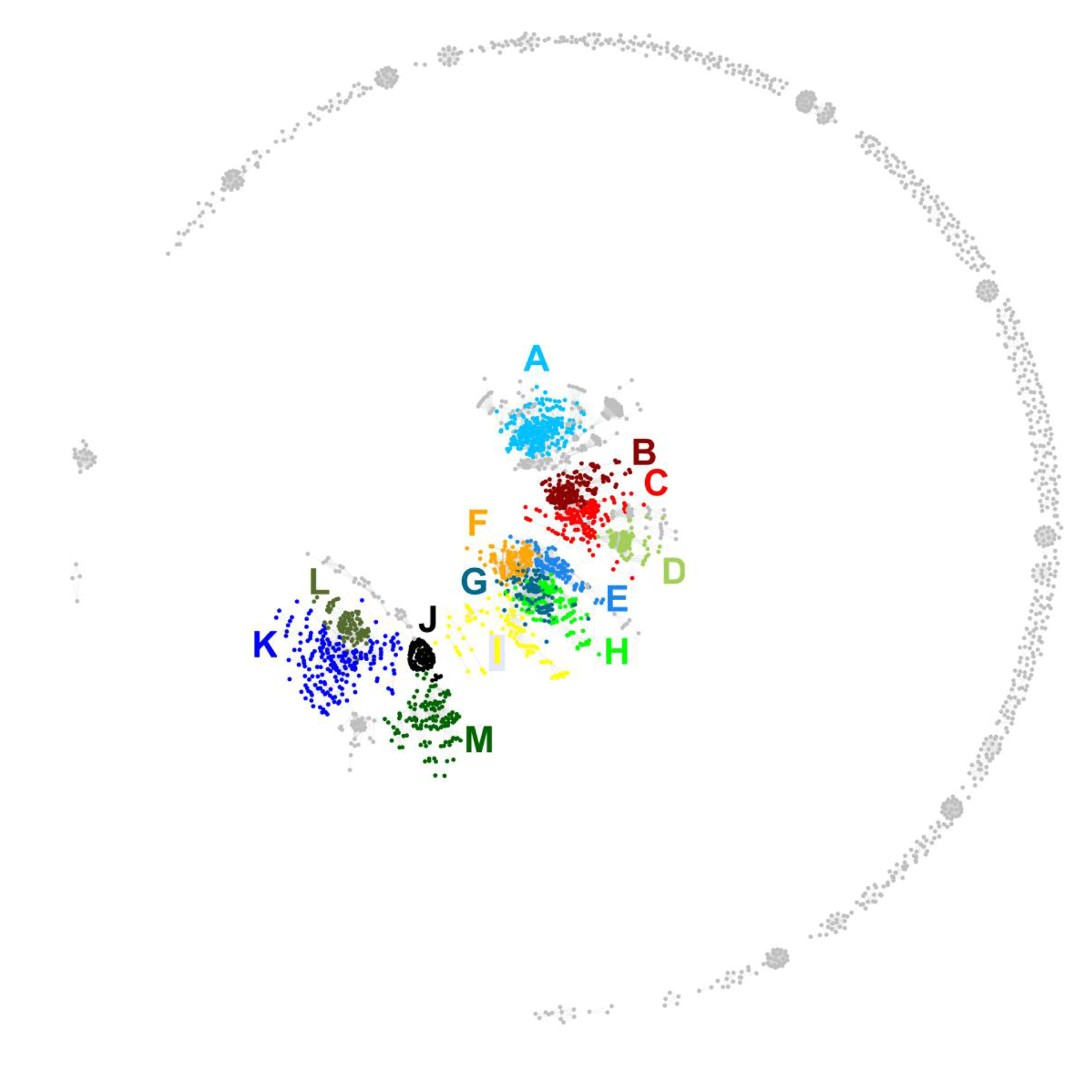

Supplement: Supplementary Figure 1 — Network graph of all 4,154 OTUs. Edges link nodes (OTUs) which have a Spearman correlation value > 0.85 across 41 optimum niche values. Color coding is according to the 13 network groups with the largest numbers of OTU. [file Image_1.TIF]

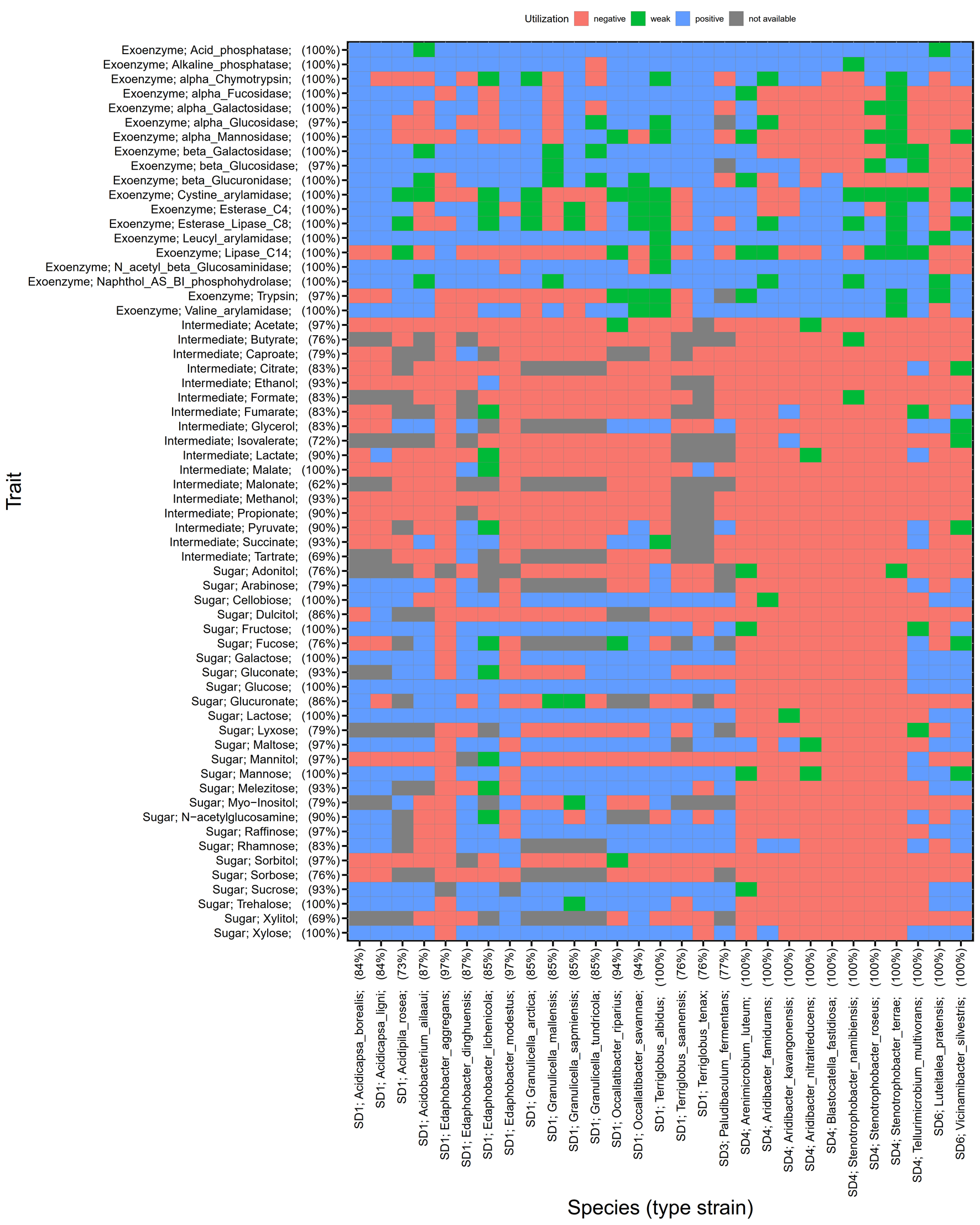

Supplement: Supplementary Figure 2 — Phenotypic properties of 29 Acidobacterial type strains. [file Image_2.TIF]

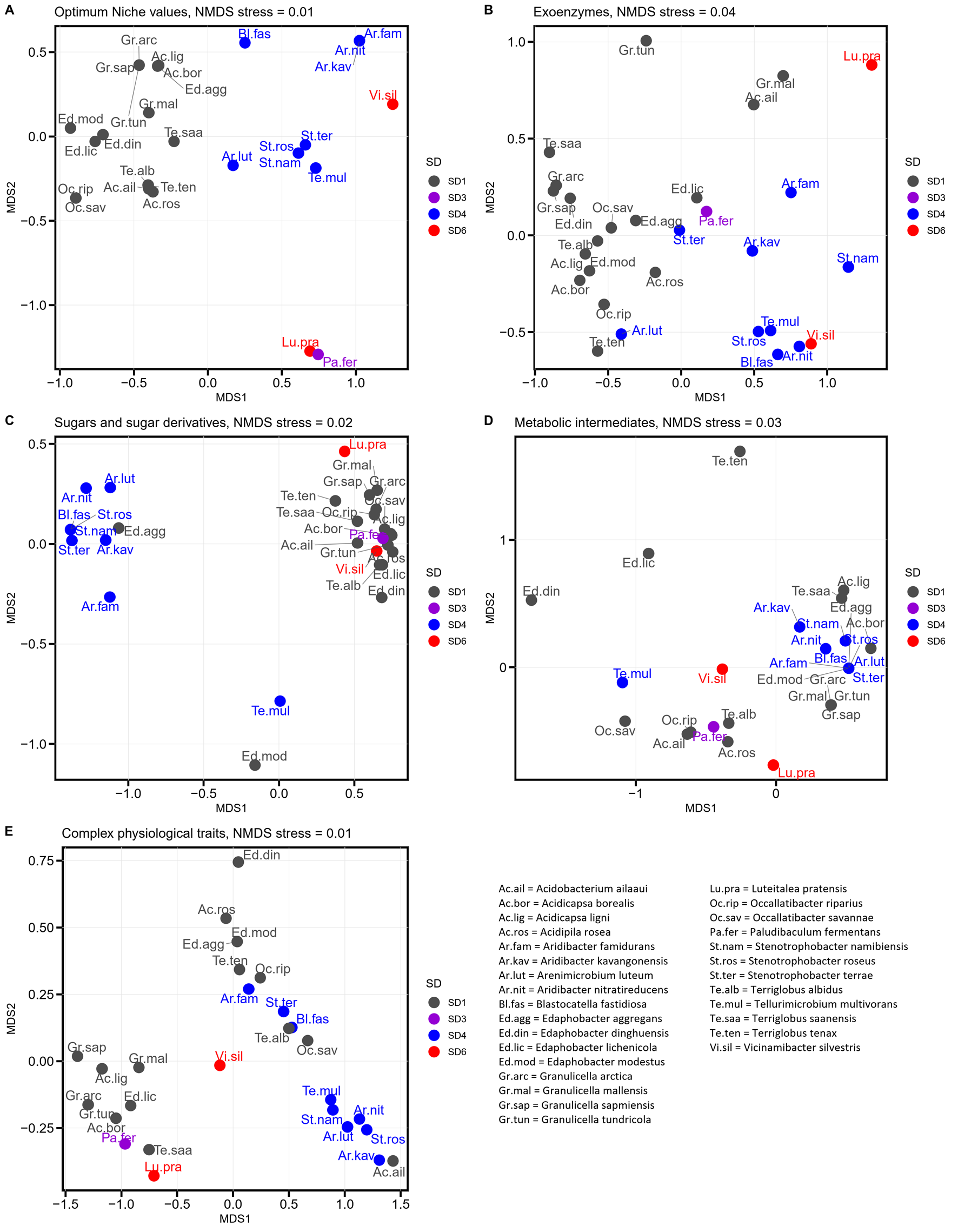

Supplement: Supplementary Figure 3 — NMDS analysis of 29 Acidobacterial type strains by 5 different trait matrices. [file Image_3.TIF]
